# Understanding the visible-light photocatalytic activity of GaN:ZnO solid solution: the role of Rh_2–*y*_Cr_*y*_O_3_ cocatalyst and charge carrier lifetimes over tens of seconds†
†Data underlying this article will be made available on the Zenodo repository at DOI: 10.5281/zenodo.1311206.
‡
‡Electronic supplementary information (ESI) available: Additional spectra and kinetic assignments, transient kinetics and spectra, stepwise H_2_ and O_2_ production, H_2_ production in different solutions. See DOI: 10.1039/c8sc02348d


**DOI:** 10.1039/c8sc02348d

**Published:** 2018-08-15

**Authors:** Robert Godin, Takashi Hisatomi, Kazunari Domen, James R. Durrant

**Affiliations:** a Department of Chemistry , Centre for Plastic Electronics , Imperial College London , South Kensington Campus , London SW7 2AZ , UK . Email: j.durrant@imperial.ac.uk; b Department of Chemical System Engineering , The University of Tokyo , 7-3-1 Hongo, Bunkyo-ku , Tokyo 113-8656 , Japan; c Center for Energy & Environmental Science , Shinshu University , 4-17-1 Wakasato, Nagano-shi , Nagano 380-8553 , Japan

## Abstract

A persistent challenge for the widespread deployment of solar fuels is the development of high efficiency photocatalysts combined with a low-cost preparation and implementation route. Since its discovery in 2005, GaN:ZnO solid solution has been a benchmark overall water splitting photocatalyst. Notably, GaN:ZnO functionalised with an appropriate proton reduction cocatalyst is one of the few particulate photocatalyst systems that can generate hydrogen and oxygen directly from water using visible light. However, the reasons underlying the remarkable visible light activity of GaN:ZnO are not well understood and photophysical studies of GaN:ZnO have been limited to date. Using time-resolved optical spectroscopies, we investigated the charge carrier dynamics of GaN:ZnO and the effect of Rh_2–*y*_Cr_*y*_O_3_ proton reduction cocatalyst. Here we show that charge trapping and trap state filling play an important role in controlling the photophysics of GaN:ZnO. We also find that electrons transfer to Rh_2–*y*_Cr_*y*_O_3_ on sub-microsecond timescales, important to reduce the electron concentration within GaN:ZnO and promote hole accumulation. *Operando* measurements showed that the water oxidation process is the rate determining process, and that the dependence of the rate of water oxidation on the accumulated hole density is similar to common metal oxides photoanodes such as TiO_2_, α-Fe_2_O_3_, and BiVO_4_. Remarkably, we show that the recombination timescale of holes accumulated on the surface of GaN:ZnO is on the order of 30 s, distinctly longer than for metal oxides photoanodes. We conclude that the unusual visible light activity of GaN:ZnO is a result of large electron–hole spatial separation due to the preferential flow of holes to the GaN-rich surface and efficient electron extraction by the cocatalyst. Our studies demonstrate that in depth spectroscopic investigations of the charge carrier dynamics of photocatalysts yield important information to understand their behaviour, and identify key properties to deliver outstanding performance.

## Abstract 2

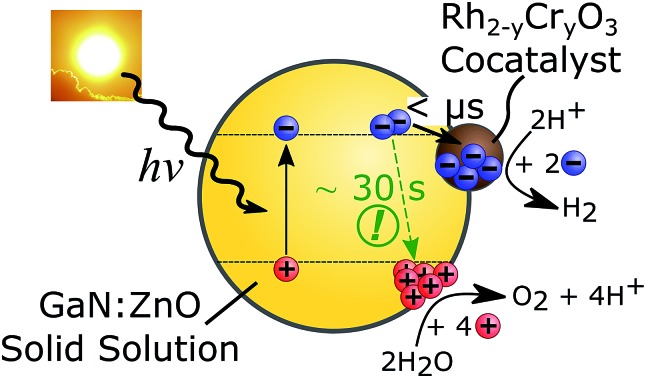
Time-resolved spectroscopies reveals remarkably long charge carrier lifetime in GaN:ZnO solid solution leading to hole accumulation key to water oxidation.

## Introduction

Photocatalytic water splitting to generate hydrogen gas as a high-energy chemical fuel is attracting significant interest as a way to decarbonise our energy supply and mitigate climate change.^1^–^3^ Many different approaches are being investigated for solar-driven H_2_ generation, such as photoelectrochemical cells,^4^,^5^ dye-sensitized photoelectrosynthesis cells,^6^–^8^ photovoltaic and electrolyzer combinations,^9^ and particulate photocatalysts.^10^,^11^ Particulate photocatalysts are pursued as a way to significantly reduce overall costs by removing the need for electrical connections and the ease of scale up. Photocatalyst panels can easily be made from particle slurries and are poised for commercial development.^12^–^14^ Advances in membrane separation technologies have alleviated concerns of oxygen and hydrogen gas separation.^15^,^16^ The most pressing issue with today's photocatalytic particulate systems is that relatively low efficiencies are obtained^11^ compared to leading photovoltaic and electrolyzer or photoelectrochemical approaches.^4^,^17^ While recent progress in particulate photocatalyst sheets are noteworthy,^12^,^13^ efficiencies still need further improvement in order for widespread deployment of low-cost particulate technologies.

A solid solution of GaN:ZnO^18^ is one of the few examples of stable visible-light driven overall water splitting on a single photocatalyst particle.^19^–^21^ The solid solution is typically prepared by nitriding a mixture of Ga_2_O_3_ and ZnO under NH_3_ flow at high temperatures.^19^,^22^ As bare particles, GaN:ZnO can photocatalyse the water oxidation half-reaction, yet is unable to reduce protons to generate H_2_ even in aqueous methanol solution.^23^ Deposition of a proton reduction catalyst is thus needed to enable proton reduction and overall photocatalytic water splitting.^24^ The oxygen-insensitive Rh_2–*y*_Cr_*y*_O_3_ proton reduction cocatalyst^23^,^25^ deposited on GaN:ZnO has been shown to give an remarkably efficient system, with an apparent quantum yield (AQY) of 5.1% at 410 nm.^26^ Nanostructuring further improved the AQY to 17.3% at 400 nm.^27^ The AQY is much lower than wide band gap UV-absorbing systems such as NaTaO_3_:La,^28^ Al:SrTiO_3_ (ref. 29) and Ga_2_O_3_,^30^ yet the substantial activity in the visible spectrum indicates considerably lower energy loss and that multi-electron charge transfer reactions (*i.e.* water oxidation and proton reduction) proceed with relatively low overpotentials on GaN:ZnO/Rh_2–*y*_Cr_*y*_O_3_.

The band gap of GaN:ZnO solid solution (∼2.6–2.8 eV for GaN-rich compositions) is significantly lower than that of either constituents, GaN (3.4 eV) or ZnO (3.2 eV). This has led to investigation of its electronic structure and the nature of the visible light absorption. The band structure of GaN:ZnO is typically described as the conduction band (CB) minimum formed of Ga 4s, 4p states and the valence band (VB) maximum mainly formed of N 2p orbitals followed by contributions from Zn 3d and O 2p orbitals.^19^,^31^ Band gap narrowing has previously been proposed to be due to repulsion between Zn 3d and N 2p states^19^ or a transition from occupied Zn acceptor levels to the conduction band,^32^,^33^ although a detailed structural investigation rather suggests interactions at the GaN/ZnO phase boundary that lowers the CB edge.^34^ A type I band alignment has been proposed, where the GaN phase CB and VB levels are found between those of ZnO.^34^ Notably, it was concluded that there was a much stronger VB offset (1.6 eV) between GaN and ZnO phases compared to the CB offset (0.2 eV). The photoluminescence (PL) of GaN:ZnO shows two main bands at *ca.* 480 and 650 nm (2.6 and 1.9 eV), with the lower energy emission band becoming dominant at higher ZnO content and lower excitation intensities.^33^ In analogy to GaN,^35^,^36^ photoluminescence has been assigned to donor–acceptor pair recombination between shallow donor levels (O_N_ substitution) under the CB edge with Ga (480 nm band) or Zn (650 nm band) acceptor levels above the VB edge.^32^,^33^,^37^

The structural and elemental composition of the GaN:ZnO solid solution is highly dependent on factors such as nitridation temperature and time,^31^,^34^,^38^ the morphology^22^ and choice of starting materials,^27^,^39^ and the extent of Zn volatilisation.^40^–^42^ The prepared materials show total Ga/N and Zn/O ratios that can deviate from 1, suggesting structural inhomogeneity.^19^,^43^ Furthermore, Zn volatilization during synthesis has been shown to yield Ga-rich surfaces^31^ and increase structural disorder,^40^ and inhomogeneous elemental distributions have also been observed.^22^,^44^,^45^ As a result of the energetic preference for the valence-matched nearest neighbor pairs (Ga–N and Zn–O), significant short range order is predicted^46^ and observed,^34^,^38^ which has been shown theoretically to impact the electronic properties in addition to structural properties.^46^ Experimental investigations have also proposed the formation of domains with different chemical compositions and heterojunctions within GaN:ZnO.^34^,^42^ As the precise atomic arrangement and distribution influences the electronic properties of the material, it can be expected that compositional heterogeneity plays a significant role in the charge carrier dynamics of GaN:ZnO.

The limited number of photocatalytic materials that show relatively efficient visible-light overall water splitting suggests that the GaN:ZnO/Rh_2–*y*_Cr_*y*_O_3_ photocatalyst possesses unusual properties. To increase our understanding of this remarkable system, we studied the charge carrier dynamics of GaN:ZnO and the changes in these dynamics induced by deposition of Rh_2–*y*_Cr_*y*_O_3_ using optical time-resolved spectroscopic techniques. In addition to *operando* measurements during active water splitting in H_2_O, electron scavenging and excitation intensity experiments gave us insights into the nature of the photogenerated charges and the charge recombination mechanisms. Overall we find that Rh_2–*y*_Cr_*y*_O_3_ is efficient at extracting electrons from the conduction band (CB) of GaN:ZnO, enabling accumulation of high concentrations of photogenerated holes with long lifetimes on the order of tens of seconds. In combination with inherently slow electron–hole recombination, proposed to be caused by spatial inhomogeneity of the chemical composition, the high activity of GaN:ZnO/Rh_2–*y*_Cr_*y*_O_3_ is thus attributed to the ability to accumulate high densities of holes to drive water oxidation.

## Results

### Photoluminescence

**Fig. 1:**
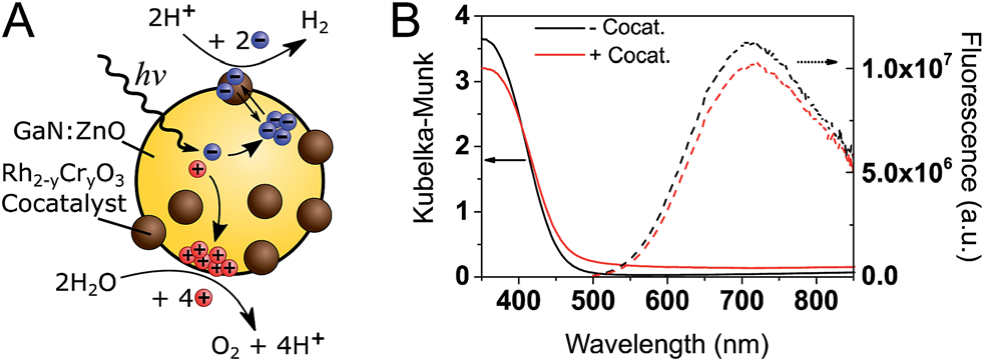
(A) Schematic of GaN:ZnO/Rh_2–*y*_Cr_*y*_O_3_ photocatalytic overall water splitting system. (B) Steady-state diffuse reflectance (left, solid lines) and PL (right, dotted lines. *λ*_ex._ = 355 nm) spectra of GaN:ZnO samples under air without (black) and with (red) Rh_2–*y*_Cr_*y*_O_3_ cocatalyst.

A schematic of the photocatalytic water splitting system is shown in Fig. 1A. Fig. 1B shows the diffuse reflectance UV-Vis absorption and the photoluminescence (PL) spectra of GaN:ZnO with and without the Rh_2–*y*_Cr_*y*_O_3_ cocatalyst. The addition of the cocatalyst results in a small increase of the absorption at wavelengths longer than 450 nm, and a 9% drop in the PL intensity. The reduction of PL intensity could be caused by charge transfer processes, although shading of the GaN:ZnO from the cocatalyst absorption is thought to play a role.

**Fig. 2:**
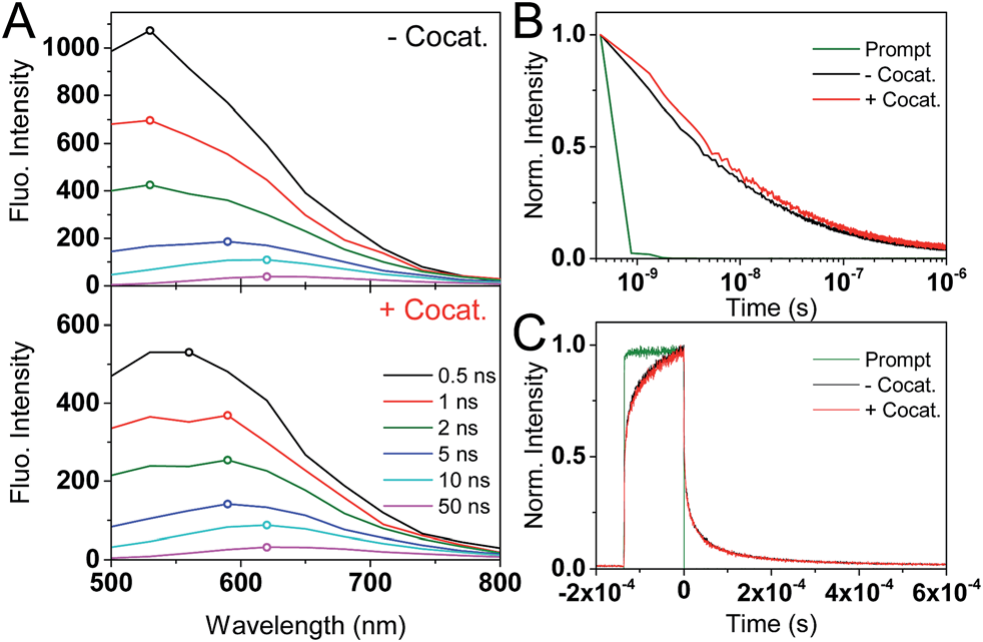
(A) Time-resolved emission spectra (TRES) of bare GaN:ZnO (top) and GaN:ZnO/Rh_2–*y*_Cr_*y*_O_3_ (bottom) in H_2_O at different time delays. The emission peak is indicated by an empty circle on each spectra. (B) tr-PL measured on the nanosecond timescale with pulsed excitation (200 ps width). (C) tr-PL measured on the microsecond timescale with short CW excitation (135 μs long). In addition to the decays for GaN:ZnO (black) and GaN:ZnO/Rh_2–*y*_Cr_*y*_O_3_ (red), the prompt (or instrument response function) is shown in green.

To understand how the photophysics of GaN:ZnO impact the photocatalytic efficiency, we begin by analysing the PL. Notably, while the absorption onset is near 460 nm (corresponding to a band gap of 2.70 eV), the emission peaks at 715 nm (1.73 eV), similar to previous reports.^33^ This emission has been assigned to donor–acceptor pair recombination between shallow O donor and deeper Zn acceptor levels.^32^,^33^,^37^ We highlight the fact that the PL maximum is found to be 1 eV lower in energy than the absorption onset. As the donor levels are thought to reside 30 meV below the CB edge, this indicates that the Zn acceptor levels are ∼1 eV above the valence band (VB) edge. Holes are thus expected to trap much deeper (*i.e.* lose more energy) compared to electrons.

tr-PL measurements (Fig. 2) show a red-shift in the position of the emission maxima over the nanosecond timescale. At early (<1 ns) times, the PL maxima is at 530 nm (2.34 eV), and shifts to 620 nm (2.0 eV) after 50 ns. A decrease in emission energy over time is indicative of charge carrier trapping, as commonly described in semiconductor quantum dots^47^,^48^ and metal oxides.^49^ No significant differences are seen between GaN:ZnO samples with or without the cocatalyst, suggesting that we only monitor the PL of GaN:ZnO.

The decay kinetics show an emission tail into the microsecond timescale (Fig. 2B). The decay is well-described by a power law of the form PL ∝ *t*^–*α*^, and an *α* exponent of 0.45 is obtained from fitting (Fig. S1†). Power law tr-PL decays imply a bimolecular recombination process, and power law exponents smaller than 1 have been observed in a range of semiconductors and have been associated with localised low energy trap states.^50^–^53^ To better probe the emission on the microsecond and longer timescales, tr-PL was acquired using continuous wave (CW) LED excitation with fast on/off switching (Fig. 2C). The long-lived PL is clearly observed, with decay times on the order of hundreds of microseconds. Importantly, the tr-PL decays on the nanosecond and millisecond timescales show dramatically different decay kinetics (Fig. S1†). We propose that this is caused by the different excitation conditions (200 ps *vs.* 135 μs pulse widths) and reflects the influence of charge trapping and spatial charge separation taking place on microsecond and longer timescales, processes which have been shown to impact the reactivity of the photogenerated charges.^54^–^57^

### Transient absorption

**Fig. 3:**
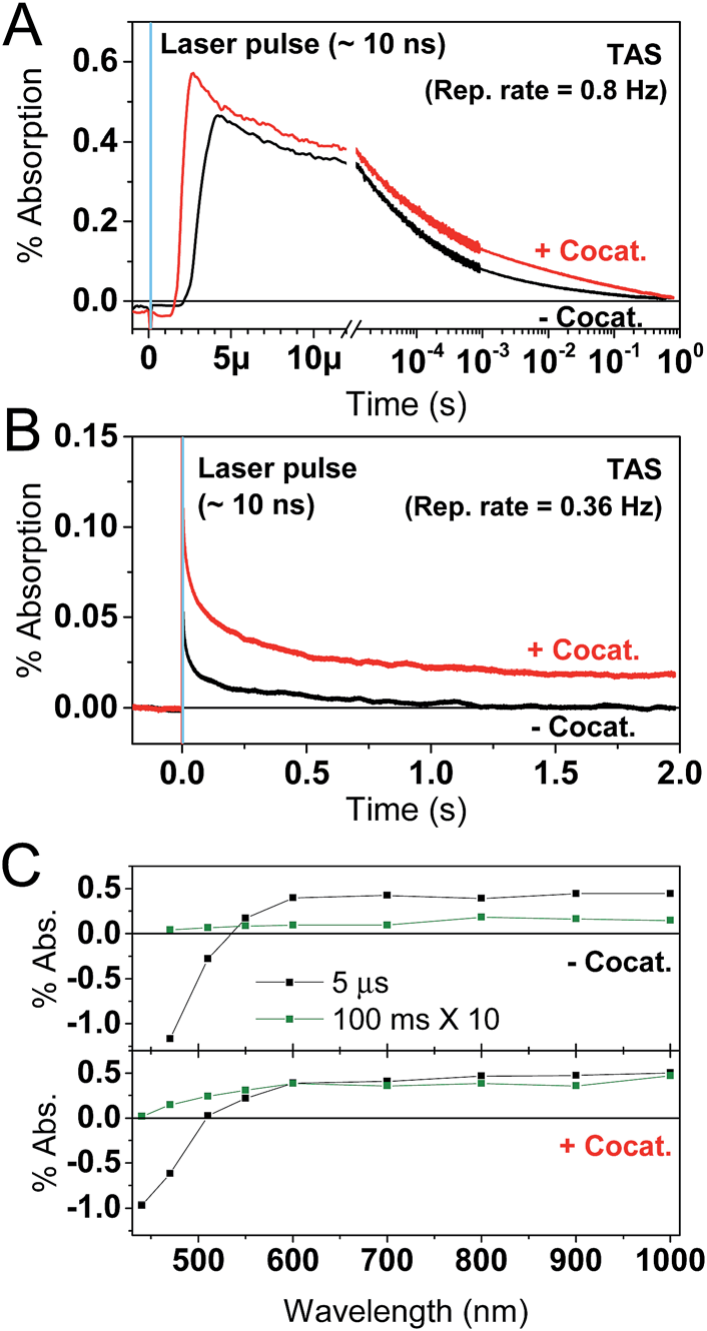
TAS results for GaN:ZnO (black) and GaN:ZnO/Rh_2–*y*_Cr_*y*_O_3_ (red) in H_2_O. (A and B) TAS kinetics monitored at 900 nm. The blue shaded areas correspond to the temporal pulse width of the 355 nm Nd:YAG laser excitation (320 μJ cm^–2^). The repetition rate of the laser excitation is indicated in the top right corner. The few μs rise of the signals in TAS is limited by the electronics and sample fluorescence. Panel A uses a linear *X* scale up to 12 μs and follows with a log 10 scale up to 1 s. (C) TAS spectra for samples without (top) and with (bottom) the Rh_2–*y*_Cr_*y*_O_3_ cocatalyst. The legend indicates the delay times for the spectra. The amplitude of the 100 ms spectra was multiplied by a factor of 10.

The PL measurements give strong evidence that charge trapping plays a significant role in controlling the photophysics of GaN:ZnO and are useful to determine the thermodynamics of trap levels. However, only emissive trap states are monitored and as emission stems from recombination, the link between PL and photoactivity is not evident. We thus turned to time-resolved absorption spectroscopies to additionally probe non-emissive trapped charge carriers commonly observed in semiconductor systems, that may yield information on the catalytically active species.^57^–^59^ Transient absorption spectroscopy (TAS) decays using a short ∼10 ns laser excitation produced a broad photoinduced absorption signal starting near 550 nm with a constant amplitude at wavelengths above 600 nm (Fig. 3; see also Fig. S2†). This resembles the reported fs-TAS spectra of GaN:ZnO powder measured in inert atmosphere which shows an increasing absorption that plateaued near 800 nm and was assigned to trapped charges.^60^ We also observed bleaching (negative transient absorption) at wavelengths < 550 nm consistent with the ground state absorption tail seen in Fig. 1B, and similar to fs-TAS measurements of Zn-rich GaN:ZnO nanoparticles in toluene that showed a bleaching signal at wavelengths < 600 nm.^61^ As detailed in the ESI,† we assign the bleaching and the spectra on the early microsecond timescale mainly to electron signal and the spectra at 1 ms and longer to be dominated by holes. While there is significant spectral overlap between the electron and hole signals over the visible and NIR ranges, the differences in lifetime are useful to distinguish both species.

The shape of the decay kinetics contains useful information regarding the behaviour of photogenerated charges. Similar to the tr-PL decays, the TAS decay kinetics of GaN:ZnO (Fig. 3) are well-described by a power law with an exponent of 0.33 (Fig. S3†), consistent with the nonexponential decays previously reported,^61^ and indicative of charge trapping–detrapping mediated recombination.^57^,^59^,^62^–^67^ Due to the dispersive nature of the kinetics, we quantify the timescale of recombination using *t*_50%_ which is defined as the time where the signal reaches half of the initial amplitude (here at *t*_0_ = 5 μs). In the absence of the cocatalyst, *t*_50%_ = 50 μs, and in the presence of cocatalyst this value increases to 80 μs. Combined with a 6% increase in amplitude in the presence of the cocatalyst, this is in line with the view that electron transfer to the cocatalyst results in increased charge separation and a larger number of reactive holes.^68^,^69^ Experiments with added Na_2_S_2_O_8_ as an electron scavenger support the idea that Rh_2–*y*_Cr_*y*_O_3_ efficiently extracts electrons on the sub-μs timescale (see ESI†). In further agreement, the effect of the cocatalyst appears to be more significant at millisecond and longer delay times, reflecting a strong influence on the hole population due to electron extraction. Reducing the laser repetition rate clearly revealed an absorbance tail up to 2 s that is only present for the sample with cocatalyst, assigned to long-lived holes (Fig. 3B). This simple observation points to slow recombination kinetics, increasing the likelihood of holes to proceed with the challenging water oxidation.

**Fig. 4:**
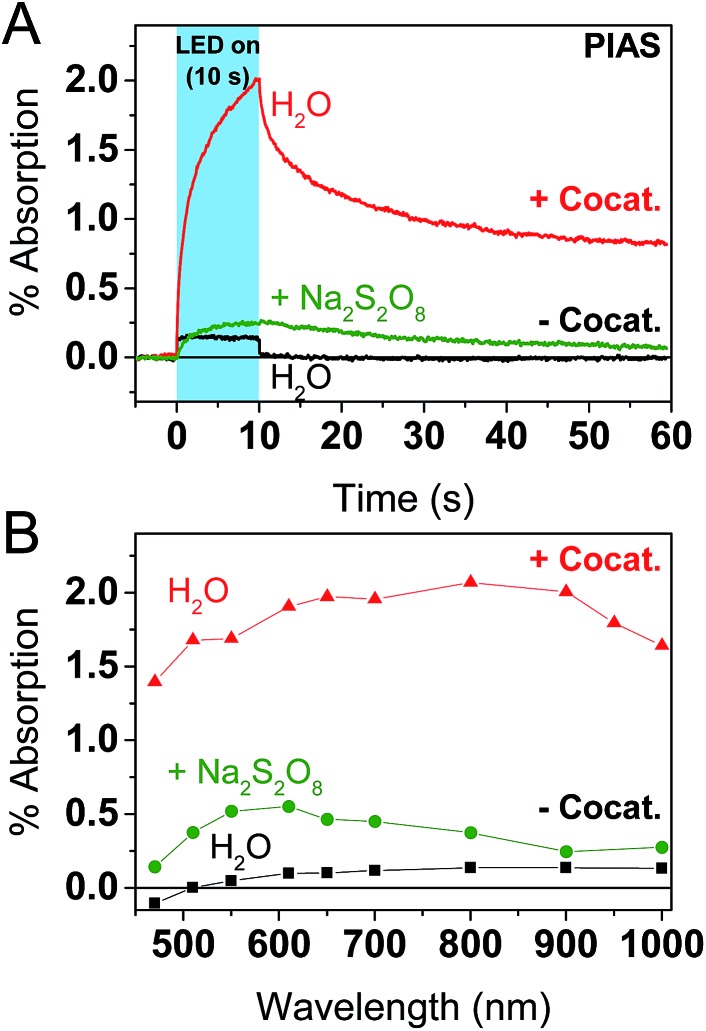
PIAS results for GaN:ZnO in H_2_O (black traces), GaN:ZnO in aqueous Na_2_S_2_O_8_ (green traces), and GaN:ZnO/Rh_2–*y*_Cr_*y*_O_3_ in H_2_O (red traces). (A) Kinetics monitored at 900 nm. The blue shaded area correspond to the temporal pulse width of the 365 nm LED excitation (10 mW cm^–2^). (B) PIAS spectra obtained following 10 seconds of irradiation. [Na_2_S_2_O_8_] = 10 mM.

To further explore the impact of the cocatalyst on charge carrier dynamics under water splitting conditions we performed photoinduced absorption spectroscopy (PIAS; Fig. 4).^70^ A CW LED is used as the excitation source and excitation pulse lengths were typically varied between 10–45 s to reach quasi-steady-state conditions. We used 10 mW cm^–2^ of 365 nm light to mimic 1 sun irradiation conditions since the incident photon flux of 1.84 × 10^16^ photon per s per cm^2^ is similar to AM1.5G conditions based on a sharp absorption onset at 460 nm (*i.e.* the integrated solar photon flux between 280–460 nm is 2.52 × 10^16^ photon per s per cm^2^). The PIAS spectra for GaN:ZnO closely resembled the initial TAS spectrum (comparing Fig. 3C and 4B), which we attribute to electron accumulation in the absence of the reduction cocatalyst; proton reduction is completely inhibited on bare GaN:ZnO whereas water oxidation does proceed.^23^ Furthermore, the growth and decay of the signal are completed within 1 s. The addition of the cocatalyst instead led to the accumulation of holes, and the slow rise and fall of the signal over tens of seconds points to kinetically slow hole processes. Remarkably, the magnitude of the PIAS signal also increased by an order of magnitude. A similar increase in amplitude and slowed down kinetics was seen for bare GaN:ZnO in the presence of the electron scavenger Na_2_S_2_O_8_, although the effect is less significant than for Rh_2–*y*_Cr_*y*_O_3_. The key role of the cocatalyst thus appears to be enabling the accumulation of high hole densities, and likely translates into a higher turnover frequency for water oxidation.

**Fig. 5:**
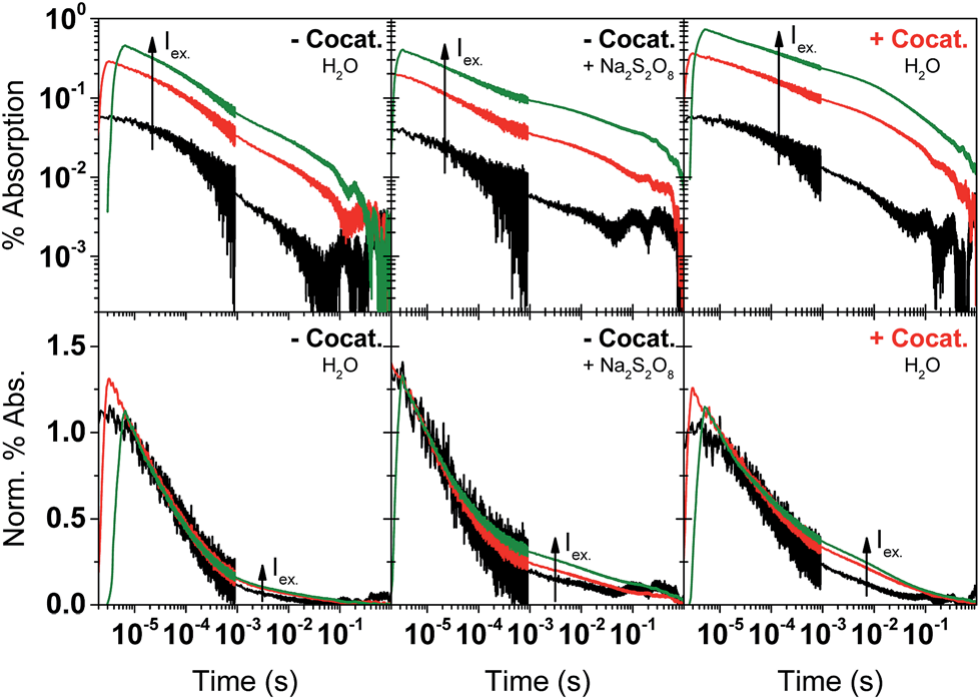
TAS excitation fluence dependence experiments for GaN:ZnO in H_2_O (left panels), GaN:ZnO in aqueous Na_2_S_2_O_8_ (middle panels), and GaN:ZnO/Rh_2–*y*_Cr_*y*_O_3_ in H_2_O (right panels). The laser excitation fluence was varied from 10 μJ cm^–2^ (black traces), to 200 μJ cm^–2^ (red traces), and 1.78 mJ cm^–2^ (green traces). Kinetics are monitored at 900 nm. The top row of panels show the data on log–log axes. The bottom row of panels show traces normalized at 10 μs on lin-log axes. [Na_2_S_2_O_8_] = 10 mM.

Excitation dependence studies gave us further information regarding the mechanism of charge recombination and trap filling. We varied the excitation fluence in TAS measurements by a factor of almost 2000, yet the change in initial amplitude is at most a factor of 12 for both bare GaN:ZnO and GaN:ZnO/Rh_2–*y*_Cr_*y*_O_3_ (Fig. 5). The decays on the microsecond timescale show parallel power law decays, and excitation fluence independent kinetics are observed on the early microsecond timescale. The differences in signal amplitude and the lack of overlap in the traces at long timescales suggest that charges do not reach a thermalized (*i.e.* Fermi-Dirac) distribution.^57^,^62^ A fast excitation dependant bimolecular recombination is inferred to take place faster than our instrument response time (on the sub-μs timescale)^60^ originating from recombination of more energetic, higher mobility carriers. On the slower microsecond and longer timescales, the amplitude of the power law decay is not seen to saturate at high laser fluences, indicating a high density of trap states that do not completely fill. Analogous behaviour has been reported in conjugated polymer:fullerene blends,^62^,^63^ carbon nitrides,^57^ and metal oxides.^59^,^64^,^65^

To better understand the effect of charge accumulation relevant to solar operation, we performed TAS experiments under an additional constant background 365 nm LED irradiation of 10 mW cm^–2^ that mimics sunlight irradiation. Faster decays (*i.e.* steeper slopes) are observed (Fig. S4†), assigned to trap filling of the lowest energy states by the background illumination.^62^,^63^ With background irradiation, no marked distinctions are made between GaN:ZnO/Rh_2–*y*_Cr_*y*_O_3_ with and without Na_2_S_2_O_8_ (Fig. S5†). For bare GaN:ZnO, the initial signal amplitude was reduced by adding Na_2_S_2_O_8_, similar to measurements without background irradiation. However, a noticeable difference is the appearance of a long-lived hole signal for bare GaN:ZnO under illumination after the addition of Na_2_S_2_O_8_. This effect can be also seen in the TAS decays without background illumination, and is amplified for the cases where electrons are extracted by Na_2_S_2_O_8_ or Rh_2–*y*_Cr_*y*_O_3_ (Fig. 5). We conclude that trap filling under higher light flux results in a greater yield of long-lived holes, promoted by electron extraction to the cocatalyst.

### *Operando* correlation of accumulated holes and water splitting rate

The PIAS results demonstrated the prevalence of hole accumulation under operating photocatalytic conditions. Hole accumulation has previously been observed in metal oxide photoanodes used for water oxidation, and plays a key role in controlling the mechanism and kinetics of the water oxidation reaction.^71^–^73^ Proton reduction (timescale ∼ μs to ms)^74^,^75^ is generally thought to be a faster chemical process compared to water oxidation (timescale ∼ ms to s),^65^,^72^,^76^,^77^ and we accordingly consider that water oxidation is the rate limiting process for overall water splitting.^78^ Mechanistic information on the rate limiting process be extracted from excitation intensity measurements since the rate law for water oxidation1
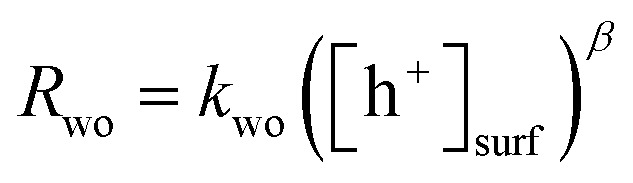
can be rewritten to give^70^,^73^
2
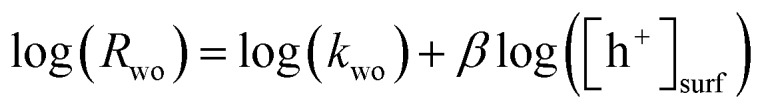
here *R*_wo_ is the rate of water oxidation, *k*_wo_ is the order-dependant water oxidation rate constant, *β* is the reaction order and [h^+^]_surf_ is the concentration of surface holes. From eqn (2) we see that the slope of the rate of water oxidation *vs.* the surface hole concentration on log–log axes yields the reaction order.

**Fig. 6:**
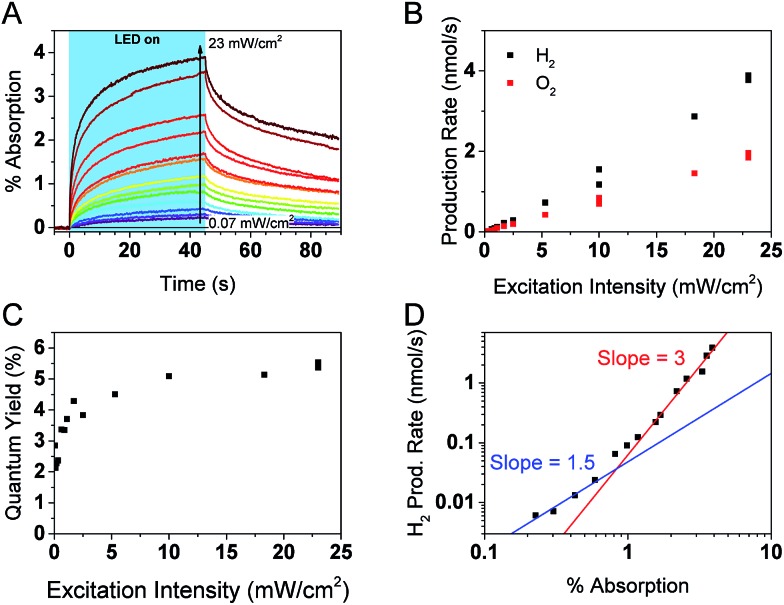
Photocatalytic and PIAS analysis of GaN:ZnO + cocatalyst samples. (A) PIAS kinetic traces monitored at 610 nm. (B) Production rates of H_2_ and O_2_ and (C) water splitting quantum yield as a function of 365 nm LED excitation intensity. (D) Rate law analysis of overall water splitting. The H_2_ production rate is plotted as a function of the optical signal observed at 610 nm after 45 s of LED irradiation at different excitation intensities, as shown in panel (A).

Simultaneous *operando* measurements of the accumulated hole concentration and the evolved O_2_ and H_2_ products are shown in Fig. 6. PIAS kinetic traces are recorded at the peak of the hole signal (610 nm) for a range of excitation intensities (Fig. 6A). A 610 nm probe wavelength was chosen to ensure that holes dominate the signal and because the large signal amplitudes accumulated over 45 s of irradiation gave a satisfactory signal to noise in a region where the probe beam is less intense. The H_2_ and O_2_ produced are measured in solution by calibrated Clark electrodes (Fig. S6†). The rate of H_2_ and O_2_ production, in the expected 2 : 1 ratio, initially appears to increase linearly with excitation intensity (Fig. 6B). However, closer inspection of the quantum yield of the water splitting reaction at each excitation intensity reveals that the process is more efficient under higher photon flux, presumably as a result of trap filling. The quantum yield is as low as 2.1% at the lowest excitation intensities, and the highest values are in the range of 5.1–5.5% at 10 mW cm^–2^ and higher irradiation intensities (Fig. 6C). These results agree well with the reported quantum yield of 5.1% at 410 nm for similar GaN:ZnO/Rh_2–*y*_Cr_*y*_O_3_ system.^26^

Fig. 6D shows a log–log plot of the rate of hydrogen production (proportional to the rate of water oxidation) against the maximal PIAS signal (proportional to the surface hole concentration). Similar conclusions are drawn when instead plotting the rate of oxygen production (Fig. S7†). We distinguish a higher slope at high hole accumulation conditions where the PIAS amplitude is >1% (corresponding to excitation intensities >1 mW cm^–2^). The slope approaches a value of 3 and supports our assumption that water oxidation is rate limiting (a maximal reaction order of 2 would be expected for the two-electron proton reduction process).^75^ As was done for the analysis of α-Fe_2_O_3_ photoanodes,^73^ we attribute the change in slope to a change of water oxidation mechanism and rate limiting step at low and high surface hole densities. While it is premature to speculate on the details of the mechanism and the intermediate species, it is interesting that the obtained slopes of ∼1 and ∼3 are the same as reported for BiVO_4_ (ref. 71) and α-Fe_2_O_3_ (ref. 73) photoanodes, suggesting similarities between water oxidation on the surface of GaN:ZnO and metal oxides. More investigations are needed to verify if this is indeed the case.

While it is clear that the PIAS optical signal is correlated with the rate of water splitting, it is not obvious whether the holes observed are directly involved in the water oxidation reaction, nor has this been determined in the limited studies of GaN:ZnO charge carrier dynamics to date. The decay kinetics of the accumulated holes after the LED irradiation is turned off are slower when higher irradiation powers are used (Fig. S8†). This is counter to previous results with metal oxides^71^–^73^ and the expectation of faster consumption of reactive holes at higher surface hole densities based on the rate law described in eqn (1). This leads us to conclude that the observed holes are not directly involved in the water oxidation reaction. Interestingly, we do observe a positive linear correlation between the amplitude of the optical signal at 610 nm and the gas production rate when different chemical scavengers are added to H_2_O (Fig. S9†). The data shown in Fig. 6D and S7† also unambiguously correlates the PIAS amplitude at 610 nm with the rate of water splitting. We rationalise these observations by considering that the holes monitored exchange with the reactive holes at steady-state conditions. As a result, the monitored hole population is an indirect probe of the reactive hole population that is directly involved in the water oxidation reaction.

## Discussion

### Role of trap states in the photophysics and photocatalysis

Both transient PL and absorbance results support the picture that charge trapping is an important process in the photophysics of GaN:ZnO. In analogy to metal oxides^55^,^65^ and conjugated polymer blends^58^ with significant density of trap states, TAS power law decays (of the form *I* ∝ *t*^–*α*^) with *α* exponents smaller than 1 are typically indicative of trapping-detrapping mediated recombination of photogenerated electrons and holes.^66^,^67^ The trapping/detrapping of charges in an exponential distribution of trap states below the band edges results in a wide range of detrapping times and recombination timescales. Trap-hindered transport of photogenerated carriers has been proposed as an explanation of activation energies insensitive to reactants,^79^ as observed for GaN:ZnO.^68^ The *α* parameter further yields information on the energetic distribution of the trap states as *E*_c_ = *k*_B_*T*/*α*,^80^ where *E*_c_ is the characteristic energy of an exponential tail of trap states. Values of *α* approaching 1 correspond to an absence of tail states and values closer to 0 correspond to a wide distribution of states. An *α* value of 0.33 is obtained from fitting the TAS GaN:ZnO decay to a power law (Fig. S3†), corresponding to an exponential characteristic energy (*E*_c_) on the order of 75 meV at room temperature. With the addition of the cocatalyst, the *α* decreases to 0.24 and results in a broader energetic distribution of states with *E*_c_ = 105 meV. The change in energetic distribution of trapped charges may be caused by the formation of sub bandgap states at the GaN:ZnO/Rh_2–*y*_Cr_*y*_O_3_ interface or a broader distribution of charges remaining in GaN:ZnO due to electron extraction.

**Fig. 7:**
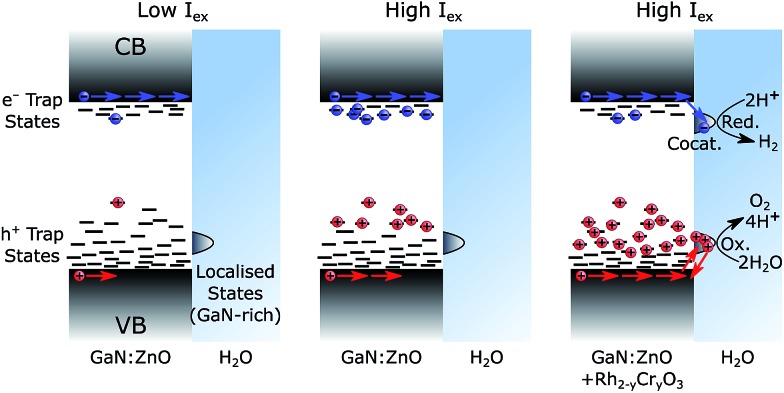
Schematic representation of trap state filling. The left case shows GaN:ZnO photoexcited with low light flux, the middle case shows GaN:ZnO photoexcited with high light flux, and the right case shows GaN:ZnO/Rh_2–*y*_Cr_*y*_O_3_ photoexcited with high light flux. Charge distribution reflects a thermalized population, as expected under continuous solar irradiation.

Confirming the presence of these trap states helps us rationalise three key observations: (1) slow charging/discharging kinetics under CW photoexcitation, (2) the larger proportions of long-lived holes when samples were subject to more light (see Fig. 5 for pulsed excitation; Fig. S8† for CW excitation), (3) the increase of the water splitting quantum yield under higher excitation densities. We ascribe these results to filling of hole trap states which results in more holes reaching the surface, as schematically represented in Fig. 7. Under low light flux, the photogenerated charges localise and trap in low energy states. We expect less significant trapping of electrons compared to holes, in line with the donor (∼30 meV) and acceptor (∼1 eV) binding energy levels based on the PL behaviour,^32^,^33^,^37^ and the fact that a portion of photogenerated electrons can reach the surface of GaN:ZnO and be extracted by aqueous Na_2_S_2_O_8_ under low light flux TAS conditions (Fig. S10†). At higher light flux and higher photogenerated charge concentration, filling of trap states results in accelerated sub-μs recombination both due to the increased charge concentration and a lower activation barrier for detrapping. However, the latter will also increase charge mobility as charges will spend more time in a high energy, high mobility state, and accordingly more holes will be able to migrate to the GaN:ZnO surface to produce the long-lived photoinduced signal probed over the visible range. Electron extraction by Rh_2–*y*_Cr_*y*_O_3_ (or similarly by Na_2_S_2_O_8_) decreases the concentration of electrons near the GaN:ZnO surface allowing for more substantial hole accumulation and trap filling. The increased hole mobility and reduced recombination from electron extraction results in a higher proportion of holes that reach the photocatalyst surface, promoting water oxidation.

### Origin of high overall water splitting efficiency

A central question to understand the remarkable photocatalytic activity of GaN:ZnO/Rh_2–*y*_Cr_*y*_O_3_ is to determine what is the root cause of the uncommon visible light activity. In the context that water oxidation is typically the rate limiting process in water splitting, the question becomes: ‘What enables high rates of water oxidation on GaN:ZnO?’. While we were unable to identify a spectral signature for the active species of water oxidation, a clear observation is the remarkably slow charging/discharging kinetics under operating conditions. It seems likely that deep hole trapping on the order of 1 eV slows down kinetics related to charge migration and recombination. Along the same line, we expect that deep hole trapping also slows down the water oxidation rate, preventing it from being significantly faster than tens to hundreds of milliseconds as observed for typical metal oxide catalysts.^65^,^72^,^76^,^77^ We also point out that the VB edge of GaN:ZnO (∼+2 V *vs.* RHE)^18^,^81^ is higher than that of metal oxides such as TiO_2_, BiVO_4_, and α-Fe_2_O_3_,^5^ and likely results in slower water oxidation kinetics.

Notably, the slow charge recombination of holes accumulated on the surface of GaN:ZnO stands out from typical metal oxide behaviour. Ultrafast measurements indicate that untrapped charge carrier lifetimes of materials such as α-Fe_2_O_3_,^82^,^83^ BiVO_4_,^84^ TiO_2_,^83^ and Cu_2_O^85^ are on the order of picoseconds – nanoseconds, and GaN:ZnO appears similar to TiO_2_.^60^ Considering the charge recombination on the microsecond timescale, the *t*_50%_ of ∼50 μs for GaN:ZnO is still comparable to metal oxides.^55^,^65^,^86^ However, the 30 s recombination timescale of surface holes on GaN:ZnO/Rh_2–*y*_Cr_*y*_O_3_ accumulated under PIAS conditions at 10 mW cm^–2^ is significantly longer than the ∼1 s lifetime observed for α-Fe_2_O_3_,^73^ BiVO_4_,^71^ and TiO_2_ (ref. 72) photoelectrodes under strong positive applied bias.

An additional distinction from typical metal oxide behaviour is apparent when comparing the rate of signal decay at different charge carrier densities from TAS and PIAS experiments (Fig. S11†). It is clear that the charge carrier concentration is not the only parameter affecting the recombination rates. In particular, the rates of decay in PIAS measured over a wide range of excitation densities show similar amplitudes and time evolution despite spanning an order of magnitude in charge carrier densities. In contrast, the traces of the PIAS decay rates *vs.* PIAS signal for α-Fe_2_O_3_ photoanodes overlap for different excitation intensities.^73^ Our data also clearly shows that the TAS decay is much faster than those measured by PIAS at similar charge carrier densities (∼0.2–0.5% Abs.). When instead considering the timescale of recombination (Fig. S12†), the TAS and PIAS traces show better agreement near 1 s delay times. This comparison suggests that temporal evolution is an important charge recombination parameter. These results also highlight that the recombination process is different between pulsed laser excitation and CW excitation, consistent with the different tr-PL decays observed (Fig. S1†).

We propose that the recombination process on the second timescale is anomalously slow because of spatial separation of charges linked to the compositional heterogeneity of GaN:ZnO. Under pulsed excitation, deep trapping of holes results in a non-thermalized distribution as evidenced by the TAS excitation fluence dependence (Fig. 5). As a result, charges localise in the nearest deep trap they encounter, and the large activation energy for detrapping lowers charge mobility and prevents efficient sampling of many trap sites within its lifetime. Under CW irradiation, thermalization of the continuously generated charge carrier takes place over tens of seconds and allows the charges to explore a range of sites and preferentially fill the lowest energy states, leading to larger detrapping activation energies and longer-lived charges.

To understand the origin of spatial charge separation, we note that the band structure of GaN:ZnO has been explained in terms of a partial segregation between GaN and ZnO phases.^34^ Heterogeneity in Zn/Ga atomic ratios has previously been seen for GaN:ZnO, even within a single particle.^22^ Preferential volatilisation of ZnO at the surface was also reported,^31^ and we thus expect a GaN-rich surface (as shown in Fig. 7) with notable compositional heterogeneity. Significantly, the valence band (VB) maximum of the GaN phase is up to 1.6 eV higher in energy than the ZnO phase, giving a strong thermodynamic driving force for holes to localise in GaN domains. The CB energy offset is much smaller (0.2 eV), and electrons will have less tendency to accumulate in a single phase. If able to sample states over sufficiently long distances, holes will relax and fill low energy states found in the GaN phase, expected at the surface. Holes will thus preferentially flow toward the surface, where band bending of this n-type GaN:ZnO may also lead to further energetic driving force for surface accumulation.^68^ The spatial separation of surface holes from bulk charges, coupled with the low electron density at the GaN:ZnO surface from efficient electron extraction to Rh_2–*y*_Cr_*y*_O_3_, results in slow charge recombination and a surface hole lifetime on the order of tens of seconds. A similar spatial charge separation argument at cubic/wurtzite GaN interfaces has been invoked to explain persistent photoconductivity with a decay time of about 60 s.^87^

We determined that the accumulated hole species observed during operation conditions is unlikely to be the catalytically active species. We consider two plausible origins of the accumulated hole species. One scenario is that the observed signal arises from surface holes that relax into low energy states which lack the required driving force to drive kinetically competitive water oxidation *vs.* recombination. These holes can be considered energetically inactive. Alternatively, we may be monitoring holes that localise in GaN-rich domains away from the aqueous interface. These holes can be considered spatially inactive. Separated by ZnO-rich domains, a large energetic barrier would be present for the migration of the holes to active surface sites. In both these scenarios exchange could occur between the active species at surface sites and the energetically or spatially inactive species. Our experimental results cannot yet distinguish between these two scenarios. On the one hand, the hole trapping energy of 1 eV and the trapping/detrapping recombination kinetics are consistent with energetically inactive surface holes. On the other hand, the TAS decays and modest AQY indicate that most photogenerated charges recombine, which would occur in the bulk of the material. Further investigations are needed to identify the catalytically active oxidation species and the precise nature of the accumulated holes species. Doing so will also open the door to a deeper structural and mechanistic understanding of the surface catalysis, as shown for the case of the well-studied α-Fe_2_O_3_.^88^

## Conclusions

We have applied time-resolved PL and transient absorbance spectroscopies to elucidate the charge carrier dynamics of GaN:ZnO/Rh_2–*y*_Cr_*y*_O_3_. Many of the behaviours found for GaN:ZnO are similar to those observed in better understood semiconductor systems, namely polymer:fullerene blends and metal oxides: (1) charge trapping is an important photophysical process on the nanosecond timescale and longer, (2) trap states gradually fill under higher light fluxes, (3) the water oxidation kinetics are highly dependent on the surface hole concentration, with a change in mechanism at high hole concentrations.

Under light fluxes relevant to solar operation, we found a recombination timescale of ∼30 seconds for GaN:ZnO/Rh_2–*y*_Cr_*y*_O_3_. We highlight that this slow recombination kinetics of surface-accumulated holes clearly stands out from usual metal oxide behaviour. The lifetime of surface holes in metal oxide photoanodes is typically ∼1 s under the additional influence of a strong positive bias, and even shorter without bias. It seems likely that the surprising visible light activity of GaN:ZnO photocatalysts is related to this slow recombination, enabling the accumulation of high hole concentrations that accelerate the rate of water oxidation. We do not find any evidence of unusually fast inherent water oxidation kinetics. The sluggish charging/discharging kinetics and relatively high VB edge of GaN:ZnO make an intrinsically fast hole transfer to water (*i.e.* a fast rate constant) unlikely.

We found evidence for deep trapping of holes on the order of 1 eV, and that filling these states promotes surface hole accumulation. It stands that reducing the density of hole trap states should be a promising path to increase the water splitting efficiency. The reality might be much more complex, however, as the hole acceptor states may be linked to the visible light absorption^32^,^33^ and charge trapping reduces the rate of recombination.^89^ It remains to be seen if significant improvements can be made from trap state engineering.

Spatial separation from electron extraction to the cocatalyst on sub-μs timescales is expected to increase the hole lifetime, yet cocatalysts are commonly deposited on metal oxides without the appearance of charge carrier lifetimes on the order of tens of seconds. To explain the long lifetime of surface holes in GaN:ZnO, we conclude that an additional driving force for charge separation must be at play. We propose this is linked to the compositional heterogeneity of GaN:ZnO and its band structure. The surface of GaN:ZnO has been found to be Ga-rich and the GaN phase shown to have a much more positive VB. Similar to designed heterojunctions that improve charge carrier separation and photoactivity,^90^ we anticipate that holes flow toward GaN domains whereas electrons transfer to Rh_2–*y*_Cr_*y*_O_3_ particles. It is possible that some level of heterogeneity generally enhances functionality of particulate photocatalysts, for example by enhancing charge separation. Spatially-resolved measurements relating the structure to the charge carrier dynamics would be particularly insightful.^91^,^92^ Further investigations of the presence and role of heterojunctions on the nanoscale between GaN and ZnO phases could lead to a more complete understanding of GaN:ZnO and other particulate photocatalysts.

## Conflicts of interest

There are no conflicts to declare.

## Supplementary Material

Supplementary information
