# Supplementary material for: Understanding the visible-light photocatalytic activity of GaN:ZnO solid solution: the role of Rh2–yCryO3 cocatalyst and charge carrier lifetimes over tens of seconds
Source: Chem Sci. 2018 Aug 15;9(38):7546–55. doi: 10.1039/c8sc02348d (PMC6180316; doi:10.1039/c8sc02348d)
Supplement: Supplementary file 1 [file SC-009-C8SC02348D-s001.pdf]

Electronic Supplementary Material (ESI) for Chemical Science.
This journal is © The Royal Society of Chemistry 2018

Electronic supplementary information (ESI)

Understanding the Visible-light Photocatalytic Activity of GaN:ZnO Solid Solution: the Role of $Rh_{2-y}Cr_yO_3$ Cocatalyst and Charge Carrier Lifetimes Over Tens of Seconds

Robert Godin,[a,¶] Takashi Hisatomi,[b,†] Kazunari Domen,[b, c] James R. Durrant[a]*

[1]Department of Chemistry and Centre for Plastic Electronics, Imperial College London, South Kensington Campus, London SW7 2AZ, U.K. *E-mail: j.durrant@imperial.ac.uk

[2]Department of Chemical System Engineering, The University of Tokyo, 7-3-1 Hongo, Bunkyo-ku, Tokyo 113-8656, Japan

[3]Center for Energy & Environmental Science, Shinshu University, 4-17-1 Wakasato, Nagano-shi, Nagano 380-8553, Japan

[¶]Current Affiliation: Department of Chemistry, University of British Columbia, 3247 University Avenue, Kelowna, British Columbia, V1V 1V7, Canada.

[†]Current Affiliation: Center for Energy & Environmental Science, Shinshu University, 4-17-1 Wakasato, Nagano-shi, Nagano 380-8553, Japan

Table of Contents

Spectroscopic Assignments S2
Timescale of Electron Extraction by $Rh_{2-y}Cr_yO_3$ S2
Figure S1 .... S3
Figure S2 .... S3
Figure S3 .... S4
Figure S4 .... S4
Figure S5 .... S4
Figure S6 .... S5
Figure S7 .... S5
Figure S8 .... S5
Figure S9 .... S6
Figure S10 .... S6
Figure S11 .... S6
Figure S12 .... S7
Figure S13 .... S7
Figure S14 .... S7
Figure S15 .... S8
Figure S16 .... S8
Figure S17 .... S9
Figure S18 .... S9

Spectroscopic Assignments

As seen in Figures 3C and S2, the shape of the TAS spectra don't change in the presence of the cocatalyst. This indicates that we predominantly monitor charges residing within GaN:ZnO as opposed to those residing in $Rh_{2-y}Cr_yO_3$. In addition, no changes in decay kinetics are seen when the redox-inactive MeCN is used as solvent (Figures S13 and S14), suggesting that TAS solely monitors the recombination of charges within GaN:ZnO as opposed to interfacial charge transfer to the electrolyte. The same behaviour is seen in the PIAS decay kinetics, which were insensitive to the solvent environment (Figure S15).

The initial TAS spectra shows ground state bleaching < 550 nm which disappears at delays times longer than about 1 ms (Figures 3C and S2). We assign this spectral evolution to a change from an electron-dominated spectrum (< 1 ms) to a hole-dominated spectrum (> 1 ms). Electron scavenging experiments were performed to confirm the nature of the signals observed. Addition of electron scavenger (here 10 mM $Na_2S_2O_8$) is expected to decrease signals associated with photogenerated electrons and promote those of photogenerated holes. PIAS measurements of GaN:ZnO in the presence of $Na_2S_2O_8$ resulted in the slow growth and decay of signal during the LED irradiation (Figure 4A). After 10 s of irradiation, the PIAS amplitude is significantly higher compared to the same measurement in H_2O, particularly in the 500 – 700 nm spectral region, and no bleach is observed (Figures 4B and S16). The PIAS spectrum of GaN:ZnO in aqueous $Na_2S_2O_8$ is comparable to that seen for GaN:ZnO/$Rh_{2-y}Cr_yO_3$ in water (compare Figures S16 and S17), supporting the assignment of the broad long-lived photoinduced absorption to holes following extraction of electrons by $Na_2S_2O_8$ or $Rh_{2-y}Cr_yO_3$. In addition, the PIAS spectra in the presence of $Na_2S_2O_8$ or $Rh_{2-y}Cr_yO_3$ strongly resembles the TAS spectra at times longer than 1 ms. We attribute the variations in peak wavelength and spectral shape to different ratios of photogenerated holes and electrons.

Timescale of Electron Extraction by $Rh_{2-y}Cr_yO_3$

Electron scavenging experiments were also useful to infer the timescale of electron transfer from GaN:ZnO to $Rh_{2-y}Cr_yO_3$. TAS measurements showed that the addition of $Na_2S_2O_8$ to the aqueous phase reduced the initial signal amplitude seen with bare GaN:ZnO (Figures 5 and S10), supporting the assignment of the sub-millisecond signal to electrons. We note that the kinetics are indistinguishable with and without $Na_2S_2O_8$ (Figure S14), indicating that the electrons observed on the microsecond and longer timescales are unreactive toward $Na_2S_2O_8$ reduction, and by extension, the more thermodynamically challenging proton reduction. As such, we conclude that all reactive electrons are transferred from GaN:ZnO to $Rh_{2-y}Cr_yO_3$ on the fast sub-µs timescales. Indeed, the lack of electron scavenging by $Na_2S_2O_8$ for GaN:ZnO/$Rh_{2-y}Cr_yO_3$ samples (Figures S5B and S18) demonstrates that electron transfer to $Rh_{2-y}Cr_yO_3$ kinetically outcompetes electron scavenging. In line with these results, PIAS experiments also showed no changes for GaN:ZnO/$Rh_{2-y}Cr_yO_3$ when $Na_2S_2O_8$ was added (Figures S15 and S17).

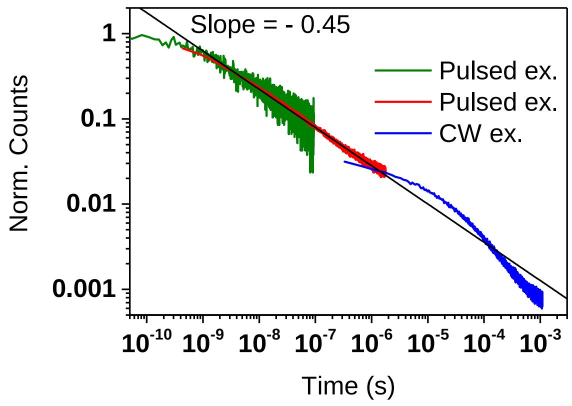

Figure S1. Comparison of the tr-PL decays of GaN:ZnO in argon purged H_2O taken with pulsed excitation (404 nm, ~ 100 pJ/cm^2) and continuous wave (CW) excitation (359 nm, 1.4 mW/cm^2), monitored at 710 – 715 nm.

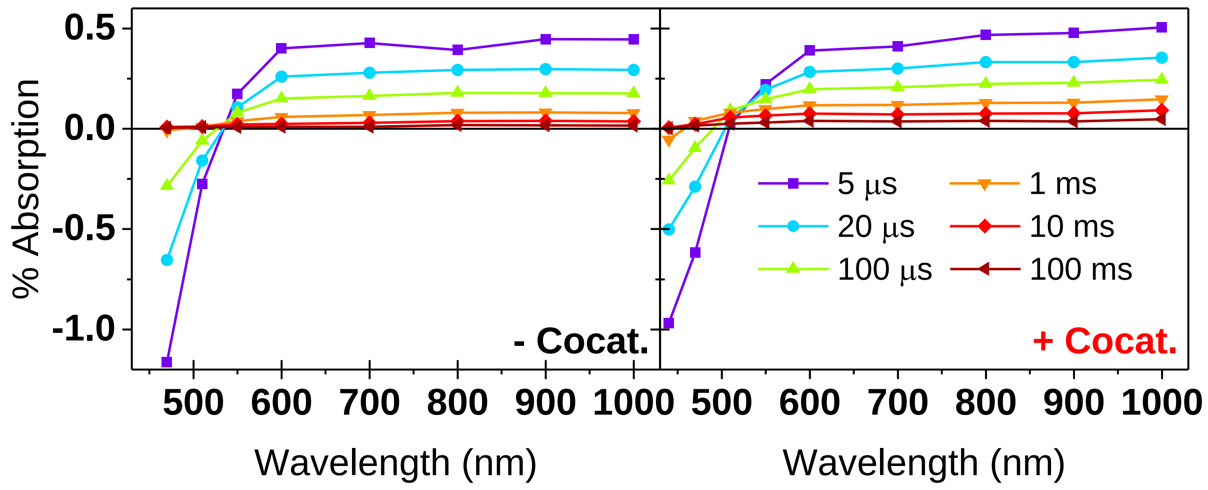

Figure S2. TAS spectra of GaN:ZnO samples in H_2O without cocatalyst (left) and with cocatalyst (right) following 355 nm laser pulse excitation (320 µJ/cm^2). Delay times are indicated in the legend.

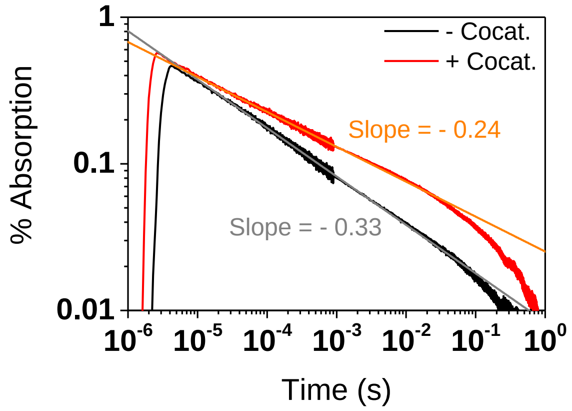

Figure S3. TAS kinetics monitored at 900 nm of GaN:ZnO (black) and GaN:ZnO/$Rh_{2-y}Cr_yO_3$ (red) in H_2O following 355 nm laser pulse excitation (320 μJ/cm^2). Data is plotted on log-log axes to emphasise the power law decay. Linear slopes correspond to the power law exponent: $I \propto t^{-\alpha} \Rightarrow \log(I) \propto -\alpha \log(t)$.

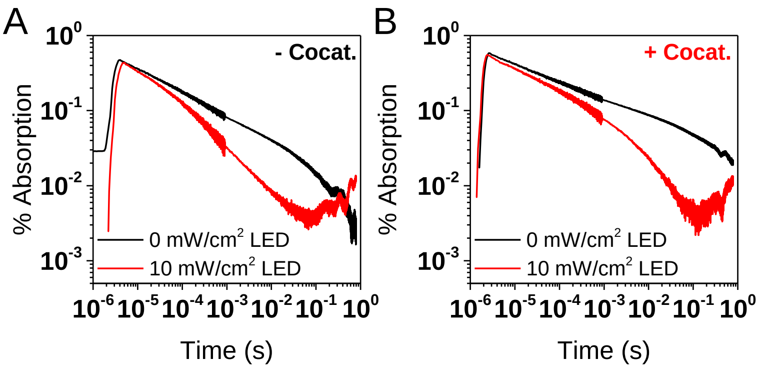

Figure S4. TAS kinetics at 900 nm of GaN:ZnO samples A) without and B) with cocatalyst in H_2O. The samples were photoexcited by 320 μJ/cm^2 355 nm laser pulses under no additional background illumiation (black) or with a constant LED irradiation of 10 mW/cm^2 at 365 nm (red).

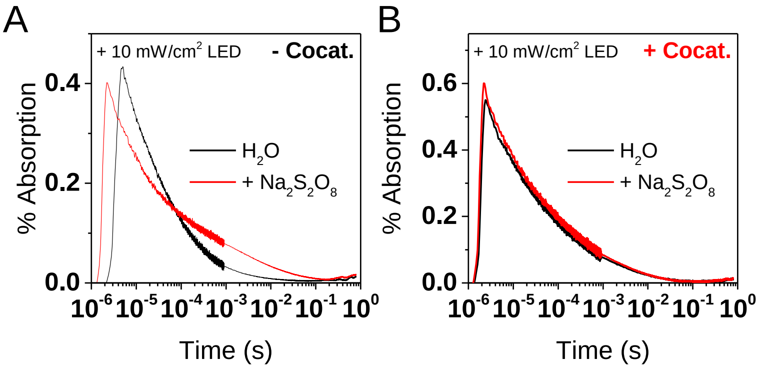

Figure S5. TAS kinetics at 900 nm of GaN:ZnO samples A) without and B) with cocatalyst in H_2O (black) and in aqueous $Na_2S_2O_8$ (red). Transients were initiated by 320 μJ/cm^2 355 nm laser pulses. The samples were also irradiated by a constant LED irradiation of 10 mW/cm^2 at 365 nm.

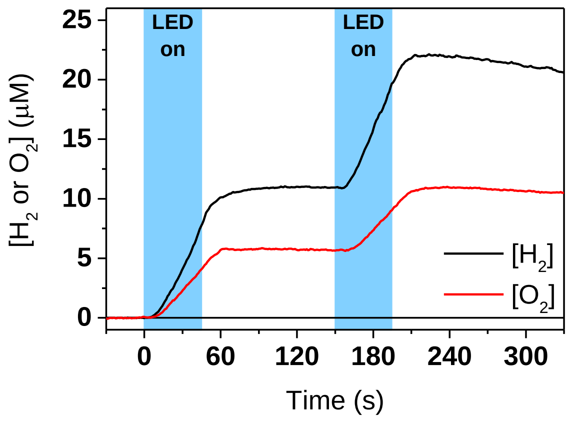

Figure S6. Calibrated Clark electrode response for H_2 (black) and O_2 (red) production under photocatalytic water splitting by GaN:ZnO/$Rh_{2-y}Cr_yO_3$. Two 45 s irradiation pulses (23 mW/cm^2) are highlighted in blue. The average step size before and after the two pulses is used to calculate the gas production rates.

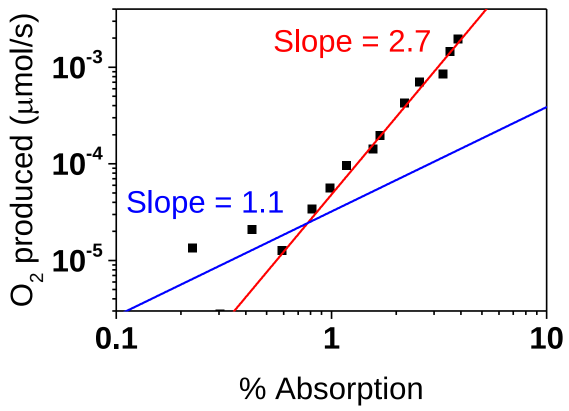

Figure S7. Rate law analysis of overall water splitting. The O_2 production rate is plotted as a function of the optical signal observed at 610 nm after 45 s of LED irradiation at different excitation intensities for GaN:ZnO/$Rh_{2-y}Cr_yO_3$ in H_2O.

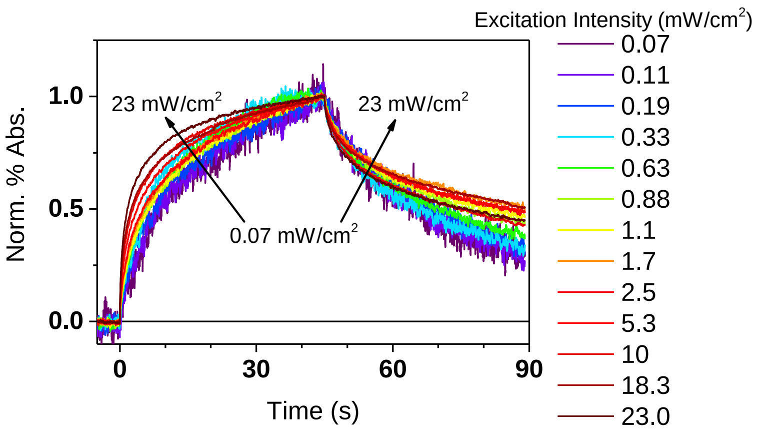

Figure S8. Normalised 610 nm PIAS traces of GaN:ZnO/$Rh_{2-y}Cr_yO_3$ in H_2O under varying 365 nm excitation intensity. Traces were normalised to the amplitude at the end of the 45 s irradiation.

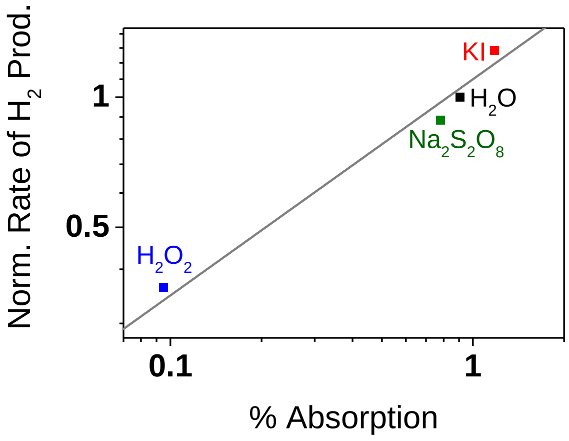

Figure S9. Correlation between the rate of H_2 production and the PIAS optical amplitude at 610 nm. GaN:ZnO/$Rh_{2-y}Cr_yO_3$ samples were submerged in H_2O (black), 10 mM KI (red), 10 mM $Na_2S_2O_8$ (green), or 10 mM H_2O_2 (blue). 10 mW/cm^2 365 nm excitation was used. Rate of H_2 production is normalised to the value obtained in H_2O.

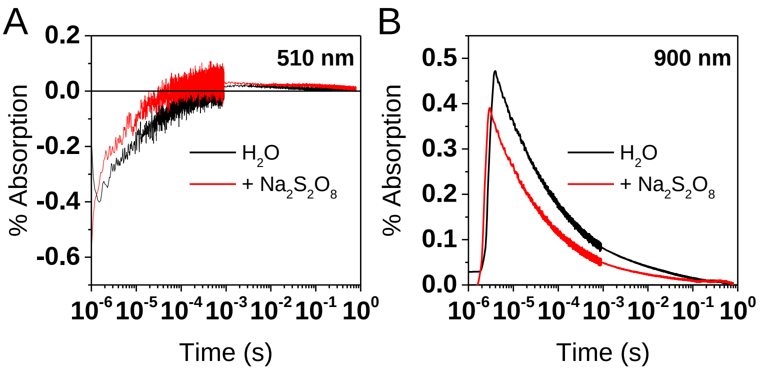

Figure S10. TAS kinetics at A) 510 nm and B) 900 nm of GaN:ZnO samples without cocatalyst in H_2O (black) and in aqueous $Na_2S_2O_8$ (red). Samples were excited by 320 μJ/cm^2 355 nm laser pulses.

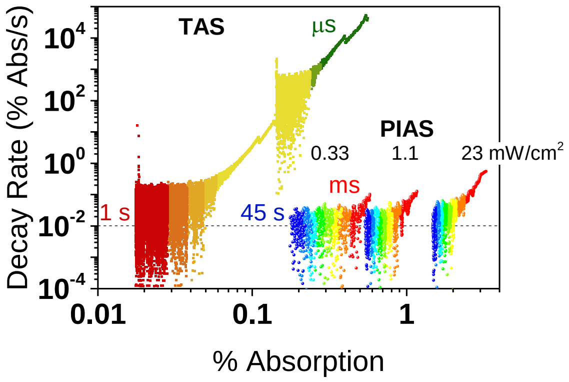

Figure S11. Comparison of the decay rates for different charge carrier densities based on the signal at 510 nm. Temporal evolution is indicated by the change in colour from green to red to blue. A dotted line is drawn at a decay rate of 1 x 10^{-2} % Abs/s.

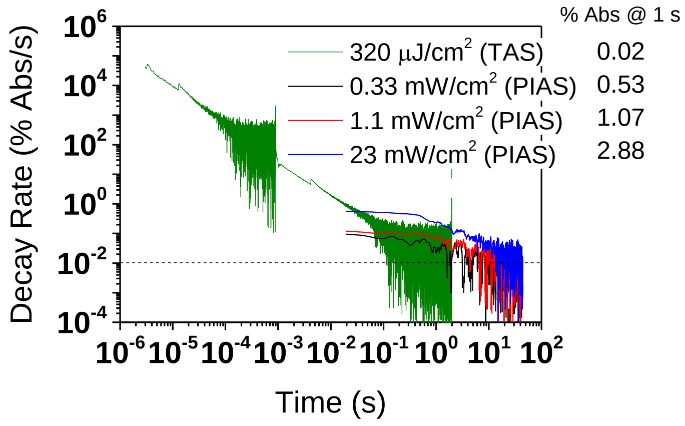

Figure S12. Comparison of the decay rates monitored at 510 nm over time. The signal amplitude at 1 s is indicated with the legend in the top right corner. A dotted line is drawn at a decay rate of 1 x 10^{-2} % Abs/s.

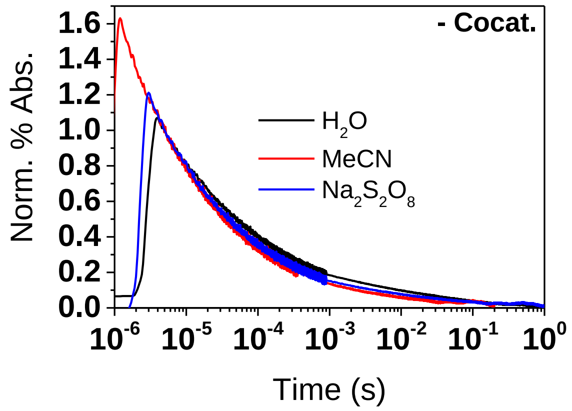

Figure S13. TAS kinetics at 900 nm normalised to the amplitude at 5 μs of GaN:ZnO samples without cocatalyst in H_2O (black), MeCN (red) and in aqueous $Na_2S_2O_8$ (blue). Samples were excited by 320 μJ/cm² 355 nm laser pulses.

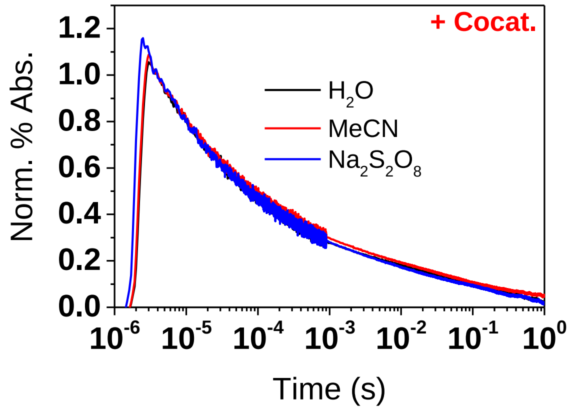

Figure S14. TAS kinetics at 900 nm normalised to the amplitude at 5 μs of GaN:ZnO/$Rh_{2-y}Cr_yO_3$ samples in H_2O (black), MeCN (red) and in aqueous $Na_2S_2O_8$ (blue). Samples were excited by 320 μJ/cm² 355 nm laser pulses.

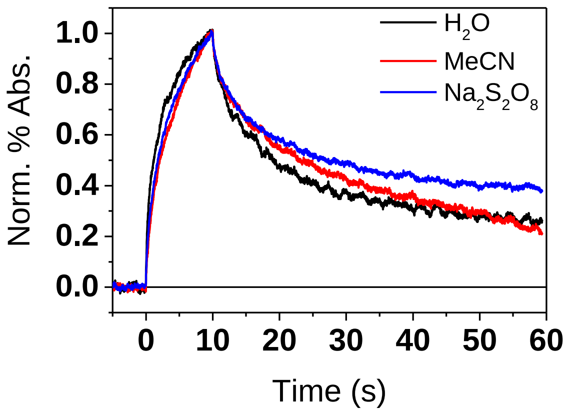

Figure S15. Normalised 610 nm PIAS traces of GaN:ZnO/$Rh_{2-y}Cr_yO_3$ in H_2O (black), MeCN (red), and 10 mM aqueous $Na_2S_2O_8$ (blue) under 10 mW/cm^2 365 nm excitation. Traces were normalised to the amplitude at the end of the 10 s irradiation.

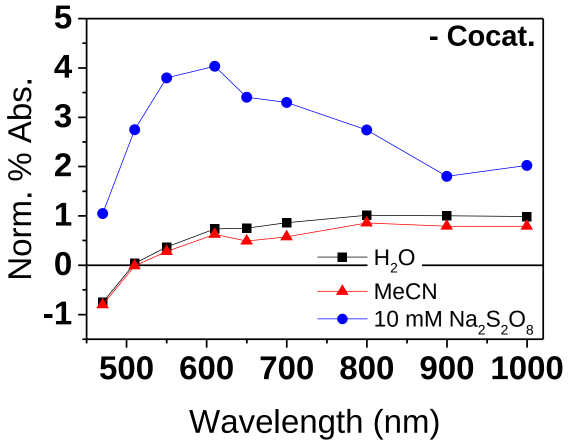

Figure S16. Normalised PIAS spectra of GaN:ZnO samples without cocatalyst after 10 s of 365 nm LED irradiation (10 mW/cm^2) for H_2O (black), MeCN (red), and aqueous $Na_2S_2O_8$ (blue) solvent conditions. Spectra were normalised to the value at 900 nm for the H_2O condition.

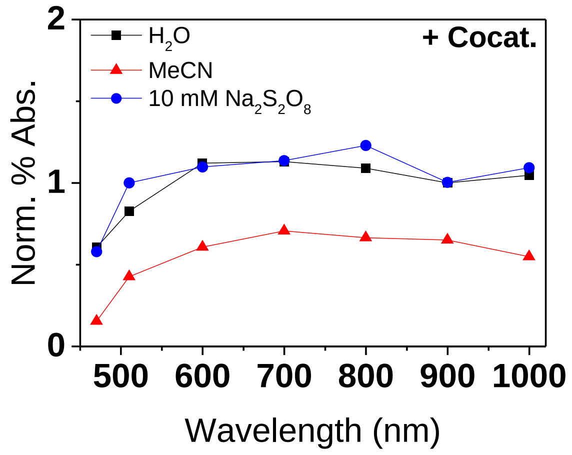

Figure S17. Normalised PIAS spectra of GaN:ZnO/$Rh_{2-y}Cr_yO_3$ samples after 10 s of 365 nm LED irradiation (10 mW/cm^2) for H_2O (black), MeCN (red), and aqueous $Na_2S_2O_8$ (blue) solvent conditions. Spectra were normalised to the value at 900 nm for the H_2O condition.

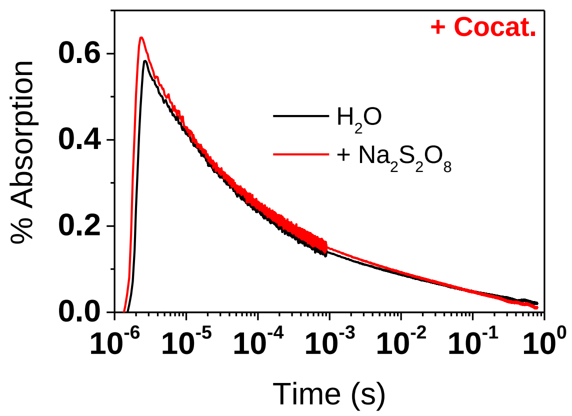

Figure S18. TAS kinetics 900 nm of GaN:ZnO/$Rh_{2-y}Cr_yO_3$ samples in H_2O (black) and in aqueous $Na_2S_2O_8$ (red). Samples were excited by 320 μJ/cm^2 355 nm laser pulses.
